# Melkersson–Rosenthal Syndrome and Migraine: A New Phenotype Associated with *SCN1A* Variants?

**DOI:** 10.3390/genes14071482

**Published:** 2023-07-20

**Authors:** Alessia Azzarà, Ilaria Cassano, Carla Lintas, Fabio Pilato, Fioravante Capone, Vincenzo Di Lazzaro, Fiorella Gurrieri

**Affiliations:** 1 Research Unit of Medical Genetics, Department of Medicine and Surgery, Università Campus Bio-Medico di Roma, Via Alvaro del Portillo 21, 00128 Rome, Italy; 2 Operative Research Unit of Medical Genetics, Fondazione Policlinico Universitario Campus Bio-Medico, Via Alvaro del Portillo 200, 00128 Rome, Italy; 3 Research Unit of Neurology, Neurophysiology, Neurobiology and Psichiatry, Department of Medicine and Surgery, Università Campus Bio-Medico di Roma, Via Alvaro del Portillo 21, 00128 Rome, Italy; 4 Operative Research Unit of Neurology, Neurophysiology, Neurobiology and Psichiatry, Fondazione Policlinico Universitario Campus Bio-Medico, Via Alvaro del Portillo 200, 00128 Rome, Italy

**Keywords:** Melkersson–Rosenthal syndrome, migraine, exome, candidate gene, *SCN1A*, precision medicine

## Abstract

Peripheral facial palsy rarely occurs as part of Melkersson–Rosenthal syndrome (MRS), which is characterized by the classical triad of tongue cheilitis, recurrent episodes of orofacial swelling, and palsy. MRS is a disorder with variable expressivity and clinical as well as genetic heterogeneity; however, the causative gene remains to be identified. Migraine is a common neurological disorder, presenting with or without aura, which may be associated with neurological symptoms. The classical example of monogenic migraine is familial hemiplegic migraine (FHM), which has phenotypic variability in carriers of variants in the same gene or even carriers of the same variant. We present a family in which two sisters displayed recurrent migraines, one of which presented recurrent facial palsy and had clinical diagnosis of MRS. We performed WES and Sanger sequencing for segregation analysis in the available family members. We identified a c.3521C>G missense heterozygous variant in *SCN1A* carried only by the affected sister. Variants in the *SCN1A* gene can cause a spectrum of early-onset epileptic encephalopathies, in addition to FHM; therefore, our finding reasonably explains the proband phenotype, in which the main symptom was recurrent facial palsy. This report also adds knowledge to the clinical spectrum of *SCN1A* alterations and suggests a potential overlap between MRS and FHM.

## 1. Introduction

Peripheral facial palsy (PFP) is a very common occurrence in neurological practice. It may manifest as unilateral or bilateral weakness or inability to move the facial muscles, which can occur suddenly or gradually, and can be temporary or permanent. In the acute phase, it can cause very marked changes in natural facial expressions, causing problems in communication and eating. PFP is a disorder with heterogeneous etiologies, ranging from idiopathic to infections, trauma, or even brain tumor. The most common form of facial palsy is the idiopathic form, called Bell’s palsy, which usually affects only one side of the face and is caused by inflammation of the facial nerve [1]. Recurrent PFP is less common and should be considered more carefully in order to exclude significant pathologies, especially when it is associated with other neurological symptoms and signs.

PFP has also been associated with other conditions such as vascular diseases and neurological disorders, such as Melkersson-Rosenthal syndrome [2,3].

One rare condition with recurrent PFP is Melkersson–Rosenthal syndrome (MRS), which is characterized by granulomatous inflammation with diffuse edema of the interstitial connective tissue and the classical triad of tongue cheilitis, recurrent episodes of orofacial swelling, and PFP [4]. However, the complete syndrome is seen only in 25% of all MRS cases; oligosymptomatic forms with non-granulomatous inflammation are frequently reported [5,6].

The cause of MRS is unknown; however, a multifactorial origin, including both a genetic basis and an environmental influence, has been proposed. The description of several families with multiple affected members provides strong evidence for a genetic basis for the syndrome [7,8].

In 1994, Smeets et al. [9] described MRS as an autosomal dominant disorder with variable expression and suggested that the defect is mapped to the 9p11 chromosomal region. Recently, through an exome sequencing approach in a single large Han Chinese family affected by MRS, Xu et al. [10] reported a heterozygous missense variant in *SLC27A1* (solute carrier family 27 member 1, OMIM *600691) as the most likely causative variant. However, this finding has not been confirmed by further reports [11] because, as most authors believe, there is likely both clinical and genetic heterogeneity in MRS and many additional causative genes remain to be identified.

An increased risk of idiopathic facial palsy (often unilateral) in migraine has been observed [12]. Migraine is a common neurological disorder presenting with or without aura, which are reversible neurological symptoms occurring before or during a migraine episode; they are most frequently visual, but also involve other senses often associated with sensory and motor disturbances. There is a strong familial aggregation for this condition, as demonstrated by twin and family studies in the 1990s [13]; in fact, the risk of migraine for a relative of an index case is about 1.5- to 4-fold compared to the general population, with an estimated heritability of about 42%. Migraine is mostly polygenic due to multiple genetic variants of minor effects that accumulate and lead to the disease. By analogy to polygenic risk score (PRS) calculations in neuropsychiatric disorders, a similar approach has been proposed for migraine research, where large genome-wide datasets have now become available. This approach provides an opportunity to investigate the shared genetic risk between known and previously unestablished co-morbidities and may lead to better and personalized treatment of migraine if used as a clinical assistant [14].

At the same time, some forms of monogenic migraines, including familial hemiplegic migraine (FHM) and migraine with aura associated with hereditary small-vessel disorders, have been identified [15]. In particular, FHM is a monogenic condition characterized by variable degrees of fully reversible unilateral motor weakness in addition to other neurological symptoms and migraine, for which several genes have been identified [16]: FHM1 (OMIM #141500), FHM2 (OMIM #602481), and FHM3 (OMIM #609634), which are respectively caused by variants in the *CACNA1A* (calcium voltage-gated channel subunit alpha1 A, OMIM *601011), *ATP1A2* (ATPase Na^+^/K^+^ transporting subunit alpha 2, OMIM *182340)*,* and *SCN1A* (sodium voltage-gated channel alpha subunit 1, OMIM *182389) genes that encode ion channels or ion transporter proteins [17,18,19]. 

Interestingly, these genes have been also associated with neurological symptoms other than migraine, such as ataxia, nystagmus, and febrile comas for *CACN1A* (responsible for FHM1), and transient blindness and early onset seizures for *SCN1A* (responsible for FHM3). The use of genome-wide association studies (GWAS) and next-generation sequencing techniques (through exome sequencing) allows the identification of both polymorphisms very frequent in the population and rare variants associated with migraine. In general, the GWAS approach makes it possible to identify single-nucleotide polymorphisms (SNPs) and/or clusters of genes in chromosomal loci that exhibit high phenotype association, with the limitation of requiring large numbers of both patients and controls. In contrast, the “candidate gene” approach by NGS techniques, through the sequencing of exome and the genome, allows the identification of rare variants of high impact on the function of genes related to that phenotype, both in complex and Mendelian diseases. Accordingly, by the whole-exome sequencing (WES) approach, variants in the PRRT2 gene were found in individuals with hemiplegic migraine and paroxysmal dyskinesia. This new locus accounts for FHM4 [20]. 

The aim of this study is to perform a WES analysis on a family with complex clinical presentation, in which recurrent facial palsy is associated with migraine as the cardinal manifestation and segregates in two affected sisters to identify rare gene variants. After exome and segregation analysis, we identified a c.3521C>G missense variant leading to the (p.Thr1174Ser) protein substitution in the *SCN1A* gene. This same variant has been previously reported in the literature in association with FHM3.

## 2. Material and Methods 

### 2.1. Clinical Presentation

The index case (II-2) was a 47-year-old woman from a Caucasian family, originating from a small town in southern Italy (1500–2000 inhabitants). She presented with recurrent episodes of migraine and PFP. At the age of 44 years, she had a first attack on the left side and few months later, the right facial nerve was also involved. Brain MRI was normal, and an oral corticosteroid treatment was started, with partial recovery. Two years later, the index case had one attack on the right side, one on the left side, and mild bilateral PFP three months later. As referred, every attack was anticipated by facial pain and dysesthesias. When II-2 was referred to our department, neurological examination was normal, except for a difficulty in opening the mouth and closing the eyes, associated with involuntary contractions in the muscles of the face and excessive tearing exacerbated by chewing. Intraorally, a fissured tongue was evident. No swelling was observed in the mouth or in the face. No history of edema in this area and in other body parts was reported. II-2 also complained about recurrent paresthesia in the left side of her body. When asked, the index case reported frequent attacks of migraine aggravated by menses. Her past medical history was unremarkable except for hypertension (treated by olmesartan 20 mg/die) and recurrent episodes of colitis (colonoscopy was negative).

Complete blood count, basic metabolic profile, thyroid function, and serum protein electrophoresis were normal. Inflammatory and autoimmunity tests, including gluten sensitivity screening, were negative. On the suspicion of Melkersson–Rosenthal syndrome (MRS), a lingual biopsy was performed, revealing lymphoplasmacellular infiltrate in the lamina propria and the dilatation of small vessels. Marked epithelial acanthosis was also present while granulomata were absent.

**Figure 1:**
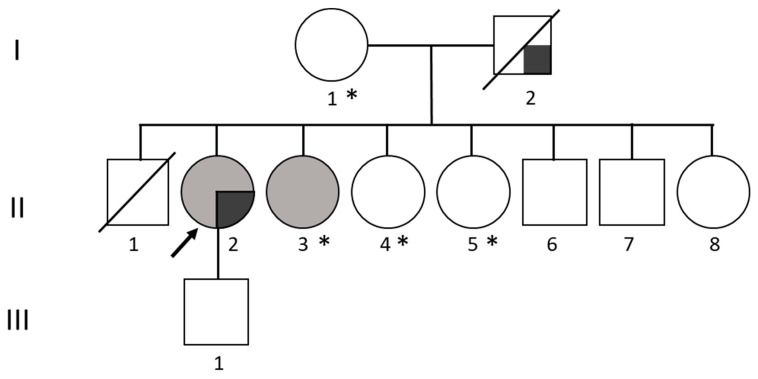
Pedigree of the family. The light grey symbols represent affected individuals with migraine; dark grey with PFP. Arrow indicates the index patient in which WES was performed. Asterisks indicate family members where segregation analysis was performed.

Through a direct interview of the index case, we obtained other pieces of information about the family history (Figure 1). The mother (I-1) was affected by type 2 diabetes and hypertension; the father (I-2) had one PFP attack but was deceased at the time of the visit. III-1 presented fissured tongue.

II-2 also has seven siblings, of which only II-3 suffers migraine attacks. This sister underwent surgery for a teratoma, and she also suffers from hyperthyroidism, colitis, psoriasis (LAC test positive), recurrent fever, and contact allergies. The brother (II-6) was affected by psoriasis and colitis. 

With respect to the other members of the family, I-1 died of liver cancer; II-4 had thyroid cancer; II-5 suffered with hypertension and hypothyroidism. Clinically, other autoimmune and/or inflammatory disease, considering negative blood tests, were likely to be excluded; considering the clinical hypothesis of MRS, the medical geneticist, in agreement with the neurologist, preferred that WES be performed.

We performed WES for individual II-2 on genomic DNA isolated from peripheral blood leukocytes and also obtained DNA from the one affected sister (II-3) as well as from two unaffected siblings and from the mother (II-4, II-5, and I-1) for segregation analysis. The mother was enrolled as the “non-transmitting parent” in this context. 

The other family members were not available for genetic testing. Peripheral blood was collected in two EDTA tubes, and genomic DNA was extracted using the QIAamp DNA Blood Mini Kit (Qiagen, Milan, Italy) following the manufacturer’s instructions. The study was approved by the local ethics committee (Institutional Review Board approval n◦ 04.21) and was performed in accordance with the Declaration of Helsinki for Human Rights. All patients enrolled in the study signed written informed consent.

The light grey symbols represent affected individuals with migraine; dark grey with PFP. Arrow indicates the index patient in which WES was performed. Asterisks indicate family members in which segregation analysis was performed.

### 2.2. Bioinformatics Analysis of WES and Sanger Sequencing

WES was performed for II-2, the affected index patient, by the service at Dantelabs, requesting an average coverage of 60X on an Illumina platform. A pair of FastQ files was delivered. Mapping, variant calling, filtering process, annotation, and variant prioritization was performed in-house according to our internal pipeline for exome analysis, which follow best practice recommendations for all bioinformatics analyses [21,22]. In brief, on the UseGalaxy online platform [23], the read files were aligned to the human reference genome assembly (GRCh37/hg19) using Bowtie2, after removing the duplicates using RmDup, variant calling was performed with FreeBayes, and the resulting VCF was annotated with wANNOVAR using different databases [24]. Then, the variant annotated file was filtered using Microsoft Excel functions. To prioritize all variants, we used VarElect software with the Human Phenotype Ontology (HPO) keywords to assess if the list of genes obtained was most likely associated with the pathogenesis of diseases. All variants in the genes were manually inspected by loading all the reads as a custom track on the UCSC Genome Browser to confirm their presence. Validation and segregation analyses of the identified variants in candidate genes were performed in other available family members by Sanger sequencing. 

Oligonucleotide primers flanking variants were designed using the Primer3 application on the UCSC genome browser. The 5′-3′ sequences of forward and reverse primers are “tcttggcaggcaacttattacc” and “caagctgcactccaaatgaaag”, respectively. Each amplicon was PCR amplified using the following standard cycling: 95 °C 5 min; 95 °C 30 s, 60 °C 30 s, 72 °C 15 s, for 34 cycles; final extension at 72 °C for 15 min. Then, 2.5 microliters of each amplicon was purified with 0.5 µL of a 1:1 mixture of exonuclease III and shrimp alkaline phosphatase at 37 °C for 15 min, followed by heat inactivation at 80 °C (ArticZymes, Tromsø, Norway).

Cleaned up PCR products were sequenced using BigDye terminator v3.1 Cycle Sequencing Kit (Applied Biosystems, Foster City, CA, USA) in a final volume of 10 µL and analyzed on a 3130 Genetic Analyzer (Applied Biosystems, Foster City, CA, USA). The electropherograms were analyzed by Sequencing Analysis v5.2 software (Applied Biosystems, Foster City, CA, USA). All steps were performed using manufacturer guidelines. 

## 3. Results

After alignment to the bioinformatics pipeline, the common variants with a minor allele frequency (MAF) > 0.01 in the gnomAD population, in the 3′ and 5′ UTR and intronic region, synonymous variants, and low quality and low coverage (<20X) variants located in non-coding regions were filtered out. 

The resulting 451 genes with 636 variant were first matched with genes found related to HPO with HP:0002076 “Migraine”, HP:0010628 “Facial palsy”, prioritized using VarElect, and each variant was manually inspected on the BAM file (loaded on the UCSC Genome Browser) [25]. We studied all variants individually, on the basis of their MAF < 0.01 in the gnomAD database, and evaluated their pathogenicity scores obtained from VarSome and the CADD score (up to 15) [26,27]. We used VarSome (https://varsome.com/, accessed on 28 April 2023) [23] to evaluate the global functional effect (“damaging” or “tolerated”) of the variants using different in silico tools: PhyloP100way, GERP, FATHMM, MutationTaster, SIFT, Polyphen. Based on the MAF and the CADD score, we selected two candidate variants: the c.3521C>G missense variant in *SCN1A* (NM_001165963.4) leading to the amino acid substitution (p.Thr1174Ser), and c.365G>A in the *ADORA1* (NM_001048230) gene, leading to the amino acid substitution (p.Arg122Gln).

**Figure 2:**
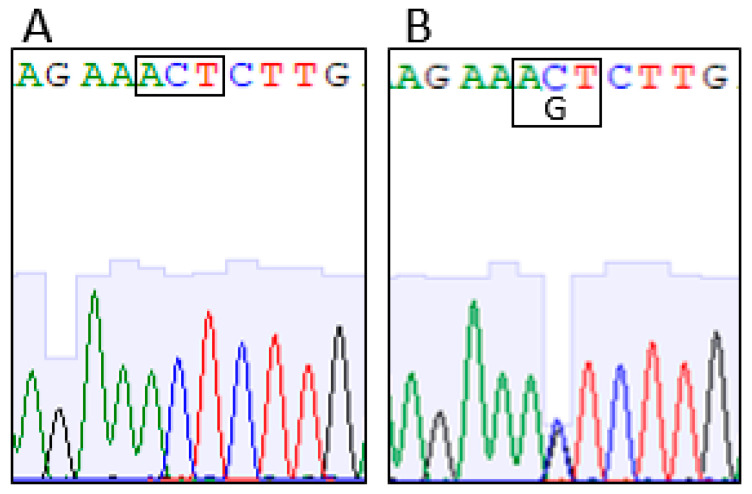
Electropherograms showing *SCN1A* variant. The codon (rectangle) and wild-type (**A**) and mutated (**B**) sequences are reported.

After segregation analysis in the family by Sanger sequencing, the variant in *SCN1A* gene was present in the affected sister II-3 but absent in the mother and in the other available siblings (Figure 2). We inferred that the variant was inherited from the father.

This substitution is rare with a MAF of 0.001706 (482/282532 alleles in gnomAD database, accessed on 28 April 2023) and a CADD score of 16.5. The gene seems to be intolerant to missense mutations, with a high Z-score (5.22) and, in fact, the transcript had fewer variants than expected. 

In UniProtKB/Swiss-Prot, this substitution (var_064309) from threonine to serine at position 1174, located in the cytoplasmatic topological domain, occurs in a highly conserved region of the SCN1A protein, in which the threonine residue is highly conserved. The PhyloP100 and GERP conservation scores are high (equal to 2.067 and 5.42, respectively), indicating a highly conserved amino acid position for Thr1174 across different species. There is not a particular change in physico-chemical properties of the amino acid (change from medium size and polar to small size and polar).

## 4. Discussion

The index patient showed recurrent episodes of PFP and fissured tongue. According to Hornstein’s criteria, this clinical condition can be classified as the oligosymptomatic form of MRS [4]. Tongue biopsy supported this diagnosis, showing non-granulomatous inflammation that is typical of incomplete forms [6].

Moreover, she suffered from migraine attacks without aura, as also did one sister, while her father had one episode of PFP and her son presented with narrowed tongue. 

According to this phenotype, the first diagnosis was MRS, also due to the fact that the patient initially did not emphasize the recurrence of migraine attacks, but was rather concerned about the PFP. For this complex clinical presentation, considering the geographical origin from a very small town in Southern Italy, where other cases of PFP had been observed, we choose to perform a WES approach. The working hypothesis was to attempt to identify a genetic variant of major impact to explain in this patient the clinical presentation of MRS associated with migraine and non-specific co-morbidities.

We started exome analysis in the index case as she was the only living family member with MRS/PFP.

After exome analysis, we found a co-segregating missense variant c.3521C>G in the *SCN1A* gene leading to an amino acid substitution (p.Thr1174Ser).

The *SCN1A* gene on 2q24.3 encodes a sodium channel α1 subunit with four homologous domains, each containing six transmembrane regions. The role of the voltage-dependent sodium channels is to regulate sodium exchange between intra and extracellular spaces and they are essential for the generation and propagation of action potentials in muscle cells and neurons [28]. 

In the ClinVar database, there are 3130 single nucleotide variants submitted for this gene, of which 2168 are missense/nonsense variations. This variant was reported in the ClinVar database as “Conflicting interpretations of pathogenicity” for sixteen submissions, of which four were submitted as uncertain significance, five as benign, and five as likely benign. Related to FHM3, and known to be associated with *SCN1A* alterations, there is one submission classified as benign because the variant was observed as part of a predisposition screen in an ostensibly healthy population and a VUS submission with affected status (SCV001369478.2).

*SCN1A* has a pleiotropic effect, since variants in this gene have been linked to several phenotypes, and heterozygous mutations in the *SCN1A* gene can cause a spectrum of early-onset epileptic encephalopathies (MIM #604403, #607208, #619317), FHM3 (MIM #609634) [29], and autism spectrum disorders (ASD) [30]. The (p.Thr1174Ser) variant was found in a sporadic patient with juvenile myoclonic epilepsy [31] and, in a second report, in a three-generation family with FHM and different epileptic phenotypes [32]. In another family, the same inherited variant was described: the proband had Dravet syndrome, while the patient’s mother suffered from frequent migraine headaches with aura but she never had seizures [33]. Along this line, the (p.Thr1174Ser) variant was found in a three-generation family in which some affected individuals had febrile seizures (FS) and/or focal occipital epilepsy while others had typical FHM or migraine with/without aura [34], and in a Tatar patient with typical clinical features of FHM, including aura and ataxia [35]. 

Taken together, the above-mentioned findings indicate that the same *SCN1A* variant can be associated with a variety of neurologic phenotypes in addition to migraine.

However, no report exists so far linking this gene to MRS.

Regarding MRS, converging evidence supports the idea that MRS is a multifactorial disease with a strong genetic basis, even if the causative genes are largely unknown. Clinically, there is a marked variability because most patients do not present all three typical features and many oligosymptomatic cases exist that are often overlooked. In familial cases, the age of onset, the disease course, and the response to treatment are highly variable among the family members [36]. Clinical heterogeneity might be explained, at least partially, by genetic heterogeneity. Similar to many other complex neurological phenotypes, it is expected that a small percentage of MRS is monogenic, whereas most instances are oligo- or polygenic. Our report suggests that one monogenic MRS is related to *SCN1A*: for the first time, we report a possible association between MRS, FHM, and the *SCN1A* gene. Interestingly, this gene encodes a voltage-dependent sodium channel that plays a key role in the generation and propagation of action potentials in neurons; thus, its alteration could produce abnormalities in nerve conduction and functionality, as observed in PFP.

However, at this time, it is difficult to definitively determine to which extent this variant in the *SCN1A* gene can cause the phenotypes we observed in the present family. Other genetic and non-genetic factors could contribute to defining the clinical picture and the extreme variability in phenotypic expression. Nevertheless, FHM is a monogenic dominant disorder with high, but not complete, penetrance. In fact, only the 70–90% of subjects with a pathogenic variant express the disease and the healthy carrier status might be related to a non-permissive genetic background.

In our family, there are two carrier sisters suffering from recurrent episodes of migraine and facial palsy, in association with other clinical features compatible with an autoimmune disease. It is very likely that they inherited the variant from their father, who had PFP or, alternatively, that he had germinal mosaicism. In this context, it turned out to be very important, after exome analysis, to characterize the patient and his/her family members in detail from a clinical point of view and collect as much as possible information regarding personal and family history, using an approach known as “reverse phenotyping”.

Finally, the role of the (p.Thr1174Ser) *SCN1A* gene variant in determining the association of recurrent episodes of migraine, PFP, and MRS remains unclear; thus, functional studies should be carried out to determine the variant-related risk. In silico modelling might also help further in understanding the biological effects of this variant, but this is beyond the scope of this study. The limitation for a strong genotype–phenotype correlation in our family is related to both the clinical and genetic heterogeneity of MRS syndrome (frequently, the triad of signs is not present), as well as to the complex nature of migraine (considering its variable clinical presentation, the wide spectrum of co-morbidities, and the multiple linked genetic loci). In addition, our study relates to only one family. Therefore, these findings need to be confirmed in more individuals and/or families in order to increase the evidence for the potential role of this candidate gene in the disease.

We believe that newly identified variants such as ours should always be described in the literature in order to assist other laboratories/clinicians in the genetic diagnosis of other patients with the same variant. In particular, the co-occurrence of PFP or MRS manifestations should be taken into account when diagnosing a patient with FMH3. The recent introduction of NGS technology in the clinical setting has substantially increased the diagnostic yield in the field of neurodevelopmental disorders and other rare and complex diseases.

The combination of NGS tools and bioinformatics algorithms for variant interpretation, in addition to in silico or in vitro modelling for pathway and compound analysis, holds great promise for precision medicine.

## 5. Conclusions

It is clear that variants in *SCN1A* are responsible for a variety of neurological phenotypes, as demonstrated by the segregation of these variants in differently affected families. In our proband, the first diagnosis was MRS based on recurrent PFP and lingua plicata, supported by tongue biopsy findings. This condition is not yet linked to a specific locus, although genetic etiology has been proposed, at least in some cases. Based on the results of exome analysis in our family, it can be suggested the *SCN1A*-related clinical spectrum may also include MRS and PFP. Our observation also shows a shared genetic profile between FHM and MRS: this is important when evaluating PRS in complex neurological traits because it suggests that the identification of variants in *SCN1A* not only increases the risk of migraine, but also of MRS. Further studies are needed to better define the genotype–phenotype correlations of the reported variant and to identify potential phenotypic modifiers. 

## Data Availability

Research data not shared.
